# Delayed Multisystem Deterioration Following Large-Volume Elvitegravir/Cobicistat/Emtricitabine/Tenofovir Alafenamide Overdose

**DOI:** 10.7759/cureus.113250

**Published:** 2026-07-23

**Authors:** Hamzah M Javed, Sebastion Cuello, Muhammad Omer, Yaroslav Buryk

**Affiliations:** 1 Internal Medicine/Critical Care, American University of the Caribbean School of Medicine (AUCSOM), Cupecoy, SXM; 2 Cardiothoracic Surgery, American University of the Caribbean School of Medicine (AUCSOM), Cupecoy, SXM; 3 Clinical Research, American University of the Caribbean School of Medicine (AUCSOM), Cupecoy, SXM; 4 Pulmonary and Critical Care, Jackson Memorial Hospital/University of Miami, Miami, USA

**Keywords:** antiretroviral overdose, case report, drug-induced cardiomyopathy, genvoya, hiv, metabolic acidosis, mitochondrial toxicity, shock requiring vasopressor support

## Abstract

Genvoya is a fixed-dose antiretroviral medication commonly used in the treatment of human immunodeficiency virus (HIV) infection and is generally well tolerated. Severe toxicity following Genvoya overdose has not been well described, and currently available reports generally describe mild clinical courses managed with supportive care alone. We present the case of a 35-year-old male with HIV, hypertension, and amphetamine use who presented after intentional ingestion of approximately 90 tablets of Genvoya in a suicide attempt. He was initially awake, alert, and hemodynamically stable. Over the next 24 hours, however, he developed distributive shock requiring vasopressor support, acute hypoxic respiratory failure requiring high-flow nasal cannula oxygen therapy, metabolic acidosis, QTc prolongation, mild acute kidney injury, and newly reduced systolic cardiac function with an estimated ejection fraction of 25-35%. Chest imaging demonstrated pulmonary edema and volume overload.

Infectious workup remained negative throughout admission. The patient was managed with supportive care in the medical intensive care unit and gradually improved over the following days. This case adds to the limited literature describing severe multisystem complications following large-volume Genvoya overdose.

## Introduction

Genvoya is a fixed-dose antiretroviral regimen containing elvitegravir, cobicistat, emtricitabine, and tenofovir alafenamide (EVG/COBI/FTC/TAF) used for the treatment of human immunodeficiency virus (HIV) infection. Although generally well tolerated at therapeutic doses, its four pharmacologically distinct components create uncertainty when substantial overdose occurs, particularly because cobicistat can alter drug metabolism through CYP3A inhibition [1]. Published reports of EVG/COBI/FTC/TAF self-poisoning remain limited and have generally described mild or predominantly metabolic abnormalities managed with supportive care [2].

The risk of delayed cardiopulmonary deterioration after large-volume EVG/COBI/FTC/TAF ingestion is therefore poorly defined. This is clinically important because patients may initially appear well despite subsequent development of hemodynamic instability, respiratory failure, or cardiac dysfunction. In addition, coexisting factors, including stimulant exposure and underlying HIV-associated cardiac disease, may complicate attribution of toxicity when drug concentrations and baseline cardiac imaging are unavailable [3-5].

We report a patient who was initially clinically stable after intentional ingestion of approximately 90 tablets of EVG/COBI/FTC/TAF but developed delayed shock requiring vasopressor support, acute hypoxic respiratory failure, metabolic acidosis, corrected QT interval (QTc) prolongation, and newly recognized severe left ventricular systolic dysfunction. This case expands the reported spectrum of EVG/COBI/FTC/TAF overdose and supports close, prolonged observation after large-volume ingestion, even when the initial presentation is reassuring.

## Case presentation

A 35-year-old male patient with a history of HIV infection, hypertension, amphetamine use, and recent abdominal liposuction presented to the emergency department after intentional ingestion of approximately 90 tablets of Genvoya in a suicide attempt related to psychosocial stressors associated with an ongoing divorce. The patient denied additional co-ingestions. Initial urine toxicology screening was positive for amphetamines. On arrival, the patient was awake, alert, and asymptomatic.

Initial vital signs demonstrated temperature 37°C, heart rate 72 beats per minute, respiratory rate 18 breaths per minute, blood pressure 105/63 mmHg, and oxygen saturation 100% on room air. Physical examination was largely unremarkable aside from anxiety. Initial electrocardiography demonstrated accelerated junctional rhythm with left ventricular hypertrophy and repolarization abnormalities.

Repeat electrocardiography later demonstrated QTc prolongation greater than 480 milliseconds. Initial laboratory studies demonstrated creatinine 1.1 mg/dL, bicarbonate 18 mmol/L, mild hypokalemia, B-type natriuretic peptide (BNP) 113 pg/mL, creatine phosphokinase 445 U/L, and arterial pH 7.25. Pertinent laboratory and clinical findings are summarized in Table 1. 

**Table 1:** Pertinent laboratory and clinical findings. CPK: creatine phosphokinase; BNP: B-type natriuretic peptide; QTc: corrected QT interval; HFNC: high-flow nasal cannula.

Investigation	Patient values	Reference values
Creatinine	1.1 mg/dL (baseline 0.8)	0.7–1.3 mg/dL
Bicarbonate	18 mmol/L	22–29 mmol/L
Arterial pH	7.25	7.35–7.45
CPK	445 U/L	39–308 U/L
BNP	113 pg/mL	<100 pg/mL
QTc interval	>480 ms	<450 ms
Ejection fraction	25–35%	55–70%
Respiratory support	HFNC required	Room air

**Figure 1:**
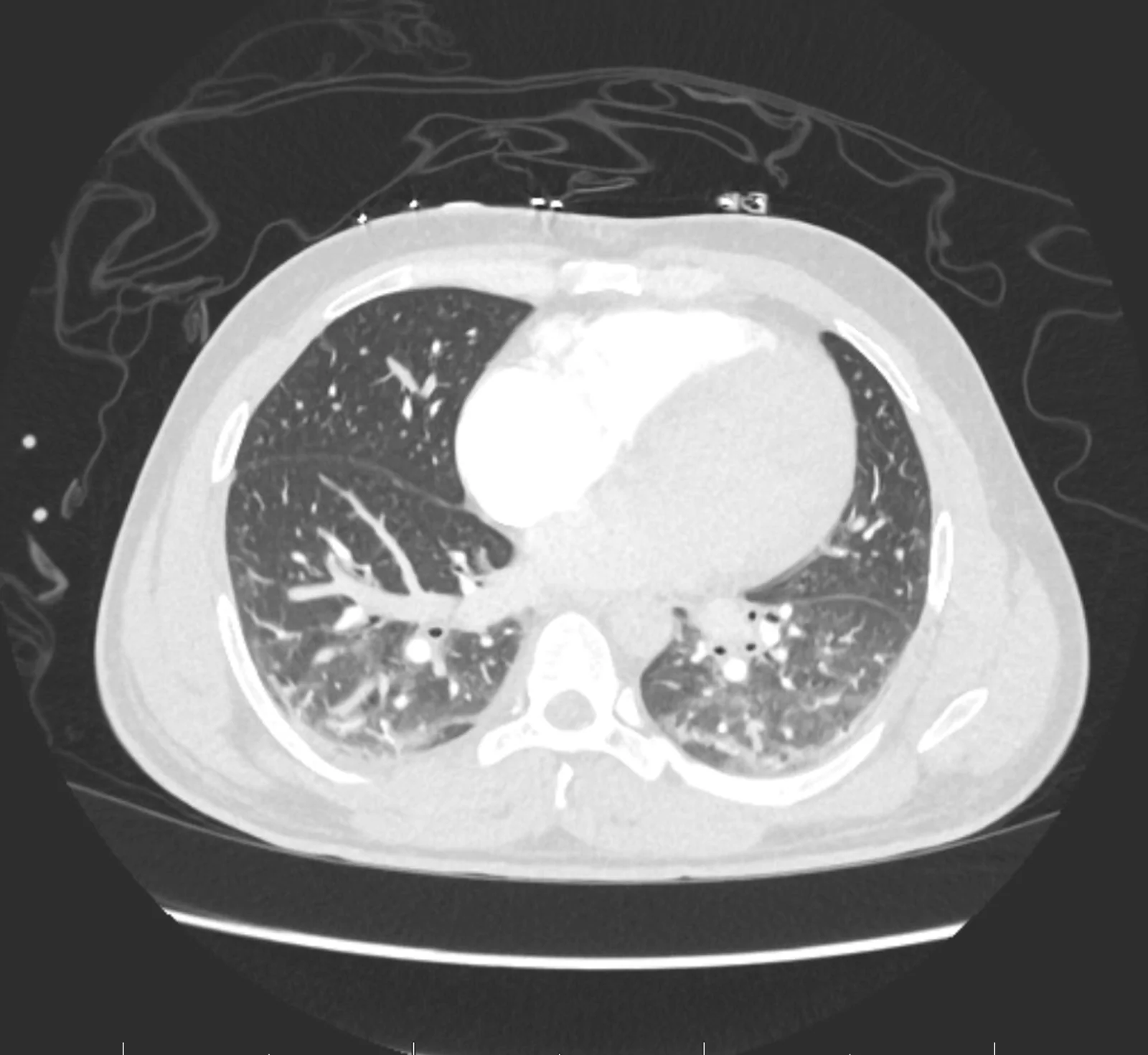
Chest computed tomography obtained after onset of hypoxic respiratory failure, demonstrating bilateral interstitial pulmonary edema and volume overload.

Lactate remained within normal limits. Poison control recommended serial blood gas and lactic acid monitoring. Quantitative serum concentrations of EVG/COBI/FTC/TAF were not obtained. Therefore, the observed clinical deterioration could not be attributed to a specific component or definitively attributed to the ingestion alone. Over the following hours, the patient became progressively hypotensive with systolic blood pressures declining into the 60s, requiring initiation of norepinephrine infusion. He subsequently developed acute hypoxic respiratory failure requiring high-flow nasal cannula oxygen therapy. Computed tomography of the chest demonstrated pulmonary edema, periportal edema, and fluid overload. Representative chest computed tomography findings are shown in Figure 1.

**Figure 2:**
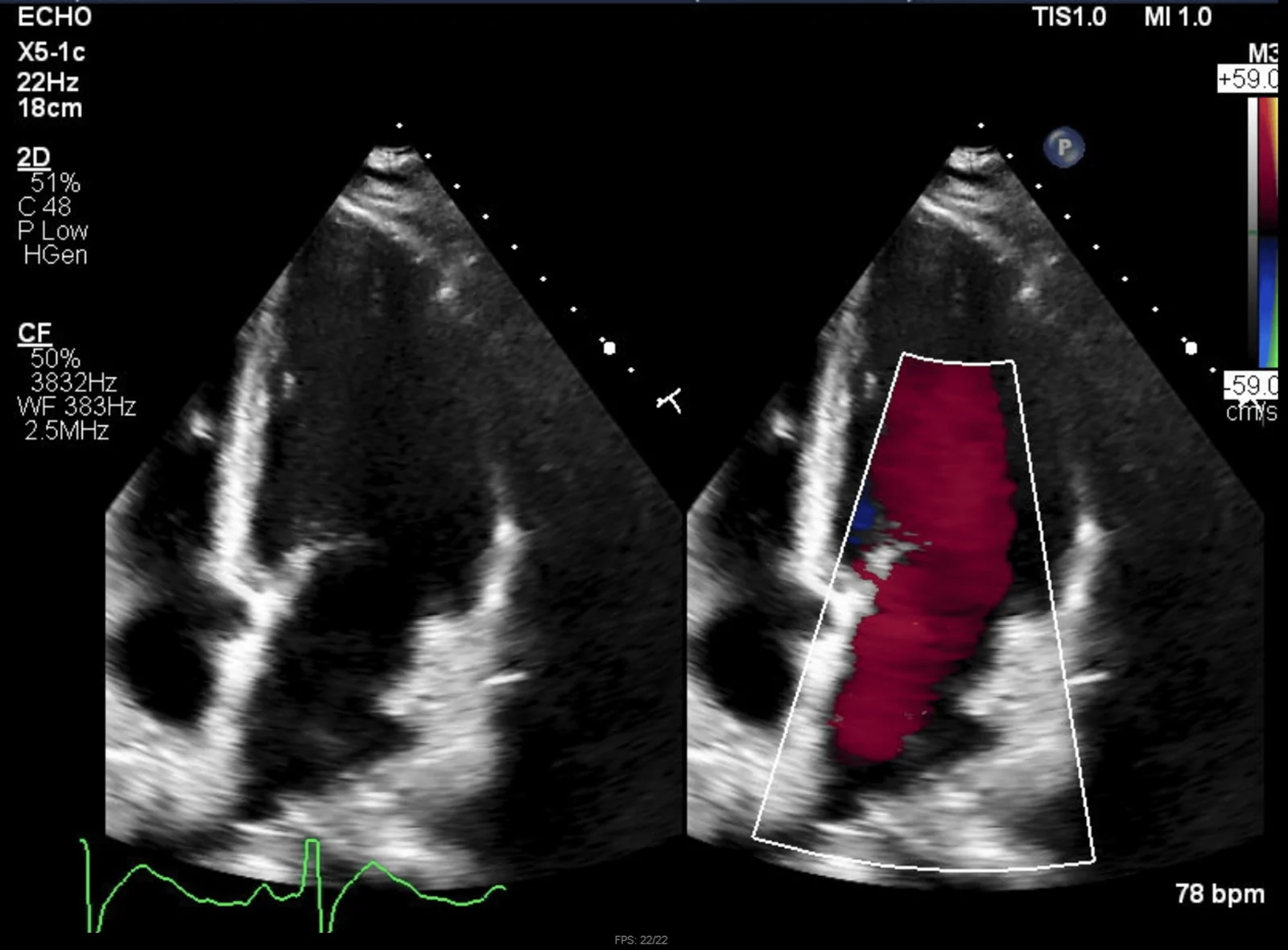
Transthoracic echocardiogram obtained after hemodynamic deterioration, demonstrating a moderately dilated left ventricle with severely reduced systolic function (estimated ejection fraction 25%-35%).

Transthoracic echocardiography later demonstrated moderately dilated left ventricular cavity size with severely reduced systolic function and an estimated ejection fraction of 25-35%. Mild right ventricular systolic dysfunction and severe left atrial dilation were also noted. Representative transthoracic echocardiographic findings are shown in Figure 2.

**Figure 3:**
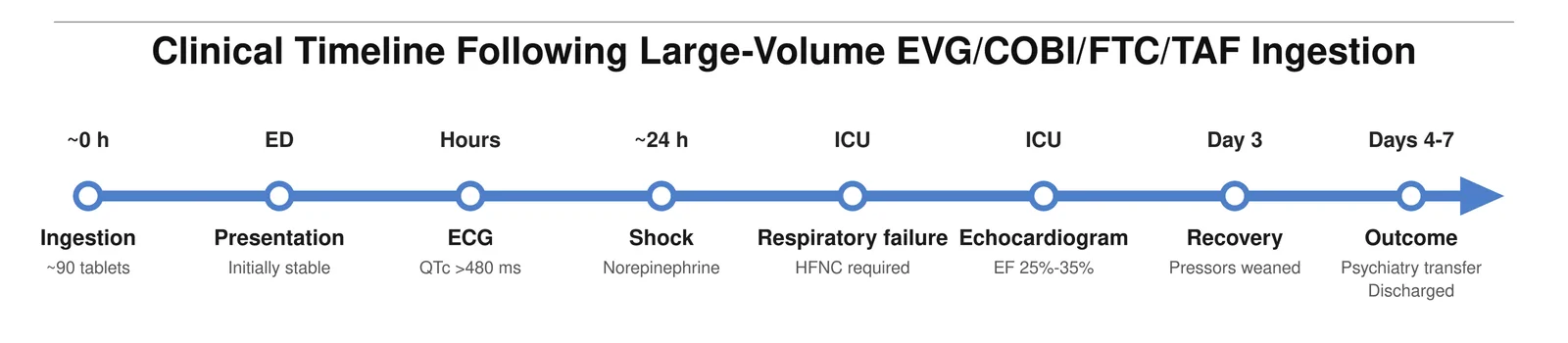
Clinical timeline showing initially stable presentation after large-volume EVG/COBI/FTC/TAF ingestion, followed by delayed hypotension requiring norepinephrine, acute hypoxic respiratory failure requiring high-flow nasal cannula, and subsequent clinical improvement with supportive care. QTc: corrected QT interval; HFNC: high-flow nasal cannula; EVG/COBI/FTC/TAF: elvitegravir, cobicistat, emtricitabine, and tenofovir alafenamide.

Infectious workup, including respiratory viral panel, remained negative throughout admission. The patient was admitted to the medical intensive care unit for ongoing supportive management. Vasopressor support and oxygen supplementation were gradually weaned over the following days. The patient became medically stable by hospital day three, was transferred to inpatient psychiatry on hospital day four, and discharged from psychiatric care on hospital day seven. The patient's clinical course is summarized in Figure 3.

## Discussion

**Figure 4:**
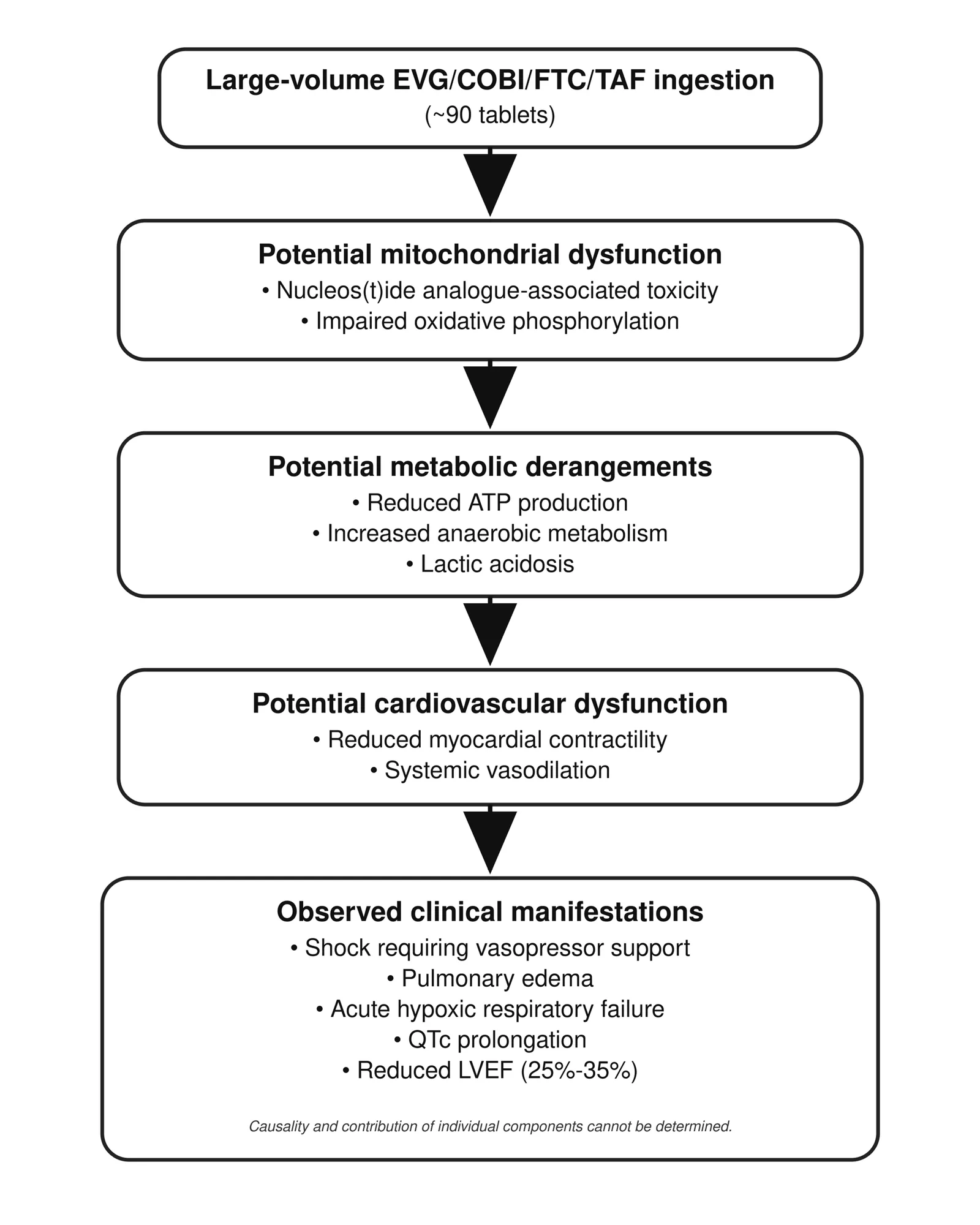
Potential contributors to delayed multisystem toxicity following large-volume EVG/COBI/FTC/TAF ingestion. This proposed model does not establish causality or identify a specific toxic component. ATP: adenosine triphosphate; QTc: corrected QT interval; LVEF: left ventricular ejection fraction; EVG/COBI/FTC/TAF: elvitegravir, cobicistat, emtricitabine, and tenofovir alafenamide.

Reports describing severe toxicity following EVG/COBI/FTC/TAF overdose remain limited [2]. Most previously reported overdoses have demonstrated relatively mild clinical courses managed with supportive care alone. In contrast, this patient developed shock requiring vasopressor support, acute hypoxic respiratory failure, metabolic acidosis, QTc prolongation, acute kidney injury, and reduced systolic cardiac function requiring ICU-level management. The fixed-dose composition of EVG/COBI/FTC/TAF complicates causal attribution because its components have distinct pharmacologic roles: elvitegravir is an integrase strand transfer inhibitor, cobicistat is a pharmacokinetic enhancer via CYP3A inhibition, and emtricitabine and tenofovir alafenamide are nucleos(t)ide reverse transcriptase inhibitors [1]. In the absence of quantitative drug concentrations, the relative contribution of each component to this patient’s clinical deterioration cannot be determined. Potential contributors to the observed multisystem toxicity are summarized in Figure 4. Mitochondrial toxicity has been described with nucleos(t)ide reverse transcriptase inhibitors; however, this mechanism could not be confirmed in this case [6].

Although chronic amphetamine use may have contributed to baseline cardiac vulnerability, the delayed onset of profound hemodynamic instability after ingestion, together with the absence of evidence for an alternative acute etiology, raises concern for overdose-associated toxicity. Prior reports have described delayed metabolic abnormalities and prolonged observation requirements following EVG/COBI/FTC/TAF self-poisoning [2]. Consistent with these reports, our patient initially appeared clinically stable before deteriorating over the following 24 hours. This pattern may support extended observation following large-volume EVG/COBI/FTC/TAF ingestion, even in patients with an initially reassuring presentation.

Cardiac findings included left ventricular dilation and an ejection fraction of 25%-35%. HIV-associated cardiomyopathy and medication-induced stress cardiomyopathy have both been described previously [3-5]. Baseline cardiac imaging was unavailable, limiting definitive attribution of systolic dysfunction to the overdose alone. Respiratory decline was likely multifactorial, including pulmonary edema, volume overload, impaired cardiac function, and systemic toxic effects. Additional reports are needed to better characterize the cardiovascular and systemic manifestations associated with severe EVG/COBI/FTC/TAF overdose.

## Conclusions

Large-volume EVG/COBI/FTC/TAF ingestion may be followed by delayed hemodynamic and cardiopulmonary deterioration despite an initially reassuring presentation. In this case, shock requiring vasopressor support, acute hypoxic respiratory failure, metabolic acidosis, QTc prolongation, and reduced left ventricular systolic function developed over the first 24 hours. Although quantitative drug concentrations and baseline cardiac imaging were unavailable, limiting causal attribution to the ingestion or a specific component, this case expands the limited literature describing severe toxicity after EVG/COBI/FTC/TAF self-poisoning. Clinicians should consider close observation for delayed deterioration after large-volume ingestion, particularly when early clinical findings are reassuring.
